# Effectiveness of Hydrocolloid Dressings for Treating Pressure Ulcers in Adult Patients: A Systematic Review and Meta-Analysis

**DOI:** 10.3390/ijerph17217881

**Published:** 2020-10-27

**Authors:** Magdalena Sylwia Kamińska, Anna Maria Cybulska, Karolina Skonieczna-Żydecka, Katarzyna Augustyniuk, Elżbieta Grochans, Beata Karakiewicz

**Affiliations:** 1 Subdepartment of Long-Term Care, Department of Social Medicine, Faculty of Health Sciences, Pomeranian Medical University in Szczecin, 48 Żołnierska St., 71-210 Szczecin, Poland; magdalena.kaminska@pum.edu.pl; 2 Department of Nursing, Faculty of Health Sciences, Pomeranian Medical University in Szczecin, 48 Żołnierska St., 71-210 Szczecin, Poland; katarzyna.augustyniuk@pum.edu.pl (K.A.); grochans@pum.edu.pl (E.G.); 3 Department of Human Nutrition and Metabolomics, Pomeranian Medical University in Szczecin, 24 Broniewskiego St., 71-460 Szczecin, Poland; karzyd@pum.edu.pl; 4 Subdepartment of Social Medicine and Public Health, Department of Social Medicine, Faculty of Health Sciences, Pomeranian Medical University in Szczecin, 48 Żołnierska St., 71-210 Szczecin, Poland; beata.karakiewicz@pum.edu.pl

**Keywords:** pressure ulcer, dressings, wound healing, meta-analysis, systematic review

## Abstract

The aim of this study was to assess the effectiveness of hydrocolloid dressings in the treatment of grade I, II, III, and IV pressure ulcers in adult patients. We compared the therapeutic effects of hydrocolloids and alternative dressings in pressure ulcer treatment. We conducted a systematic review, using a literature search only in English, from database inception until 20 April 2020, to identify randomized trials comparing various types of dressings applied in the healing of pressure ulcers. The databases were PubMed, Embase, and Cumulative Index to Nursing and Allied Health Literature (CINAHL). The study selection was performed independently by two reviewers. Data were extracted based on the guidelines included in the Preferred Reporting Items for Systematic Reviews and Meta-Analyses (PRISMA) protocol. The risk of bias in the included studies was assessed using a standardized critical appraisal instrument developed by the Cochrane Collaboration. Random-effect meta-analysis of data from three or more studies was performed using meta-analysis software (Comprehensive Meta-Analysis V3, Biostat, New Jersey, USA). A total of 1145 records were identified, of which 223 were qualified after further verification, of which eight were finally included in further analysis. Hydrocolloid dressings were not superior to control therapeutics (*p* = 0.839; Z = 0.203; CI 95%: 0.791–1.334). They were not associated with higher healing rates (*p* = 0.718; Z = 0.361; OR: 0.067; CI 95%: 0.297–0.431), nor did they decrease the incidence of adverse events compared with control therapeutics (*p* = 0.300; Z = −1.036; OR: 0.067; CI 95%: 0.394–1.333). In the above cases, Egger’s test also did not indicate publication bias (t value = 0.779, *p* = 0.465; t value = 1.198, *p* = 0.442; t value = 0.834, *p* = 0.465, respectively). The present meta-analysis shows that hydrocolloid dressings are not significantly better than alternative ones in the healing of pressure ulcers in adult patients.

## 1. Introduction

### 1.1. Description of the Condition

Pressure ulcers (PUs)—also called pressure injuries, pressure sores, decubitus ulcers, and bedsores—are localized injuries to the skin and/or underlying tissue, primarily caused by prolonged pressure and the associated tissue hypoxia or shear on the skin [1].

There are many contributors to pressure ulcer development, the main of which are mechanical forces, such as surface pressure, shear forces, friction forces, and trauma. The accompanying causes include a number of internal factors (depending on the patient’s clinical status) and external factors (independent of the patient’s clinical status but conditioned by the environment) [2]. There are many scales in the world to assess the risk of pressure ulcers and many systems describing the degree of tissue damage, classified as stage I, II, III, and IV. One of the most popular pressure ulcer classifications is that developed in 2014 by the National Pressure Ulcer Advisory Panel (NPUAP), the European Pressure Ulcer Advisory Panel (EPUAP), and the Pan Pacific Pressure Injury Alliance (PPPIA) [1].

A review of the literature shows that it is difficult to estimate the prevalence of pressure ulcers worldwide. This problem is mainly due to differences arising from their definition adopted for epidemiological and research purposes, their assessment, and data collection methods [3,4]. However, it has been estimated that in the population of the United States, pressure ulcers occur in almost 5% of people over 65 years of age, and their incidence increases with age. Around 15% of pressure ulcer cases are observed in acute care hospitals, 10% in nursing homes, and 7% in home care settings [5]. A multiservice, cross-sectional survey conducted in Great Britain showed that the mean age of people with complex wounds was approximately 70 years and that the point prevalence of complex wounds was 1.47 per 1000 of the population [6]. The research conducted at German healthcare facilities, on the other hand, demonstrated that for all people at risk of pressure ulcers, their prevalence was 21.1%, and the number of pressure ulcers per person was higher in hospitals (24.6%) than in nursing homes (13.9%) [7]. 

The incidence of pressure ulcers is determined by individual factors, depending on each patient and the type of treatment [8]. It is difficult to estimate the cost of the latter, because pressure ulcers develop in patients with various clinical conditions and numerous comorbidities that require not only local but also general treatment (e.g., protein and vitamin supplementation, antibiotic therapy) in various healthcare settings. All this means that pressure ulcer treatment is long-term, requires the use of specialized methods, and generates high costs of hospitalization, which poses a significant financial burden on the healthcare system [8]. Statistics show that in the United States, around USD 11 billion is spent annually on pressure ulcer treatment, and between USD 500 and 70,000 dollars is spent on one wound [9]. Furthermore, the therapy of patients with pressure ulcers in acute care hospitals is more expensive than in other healthcare settings [4]. 

### 1.2. Description of Interventions

Due to the global prevalence of pressure ulcers, clinical guidelines have been developed for the prevention and treatment of this health problem by leading international organizations in this field: the NPUAP, the EPUAP, and the PPPIA [1]. 

The current strategy for preventing and treating pressure ulcers consists of three main components: the assessment of the risk of pressure ulcer development, the implementation of an adequate prevention system in patients at risk of pressure ulcers, and pressure ulcer treatment. The key preventive measures are the change in pressure on the patient’s skin and the use of specialized facilities (e.g., alternating pressure mattresses), while therapeutic interventions include mainly the use of dressings and a balanced diet rich in protein, iron, vitamin C, and zinc [1]. It is also important to treat pathologies that contribute to the development of pressure ulcers, such as diabetes mellitus and hypoalbuminemia.

In accordance with the recommendations of the NPUAP, the EPUAP, and the PPPIA, pressure ulcer treatment involves various strategies, such as reducing pressure on the patient’s skin, educating patients and their families, treating pain and infection, optimizing perfusion, as well as performing surgical or chemical wound-cleaning procedures and applying dressings appropriate for the type of wound. Specialist dressings should have the following properties: ability to absorb and retain exudate, heat insulation, protection against contamination, water permeability, protection against bacteria, no tissue damage when removing the dressing, and patient comfort when changing a dressing [1].

The classification of dressings depends on the additional substances added to them, as well as on the key materials used in their production. The following properties of an ideal wound dressing are described: the ability of the dressing to absorb and contain exudate without leakage or strike-through in order to maintain a wound that is moist but not macerated; achieve freedom from particulate contaminants or toxic chemicals left in the wound; provide thermal insulation, in order to maintain the optimum temperature for healing; allow water but not bacteria permeability; optimize the pH of the wound; minimize wound infection and avoid excessive slough; avoid wound trauma on dressing removal; accommodate the need for frequent dressing changes; provide pain relief; and be comfortable [10,11].

According to the classification developed by the British National Formulary [10], the following types of wound dressing are distinguished:

- basic dressings (e.g., low-adherence dressings; absorbent dressings);
- antibacterial dressings: (e.g., honey, iodine, or silver impregnated dressings; other dressings with anti-bacterial properties);
- advanced dressings (e.g., foam dressings consisting of hydrophilic polyurethane foam; alginate dressings made of calcium and sodium alginate; hydrogel dressings consisting of cross-linked insoluble starch or carboxymethocellulose polymers and water (up to 96%); occlusive hydrocolloid dressings (HDs) consisting of a hydrocolloid matrix attached to a vapor-permeable foil or foam base; foil/membrane dressings permeable to water vapor and oxygen but impermeable to water and microorganisms; capillary action dressings consisting of an absorbent core of hydrophilic fibers contained between two weakly adhering contact layers; odor absorbing dressings, usually containing charcoal);
- specialist dressings (e.g., protease-modulating matrix dressings designed to change the activity of proteolytic enzymes in chronic wounds, which promotes natural wound cleansing) [9].

### 1.3. Objectives

The aim of this study was to assess the effectiveness of hydrocolloid dressings in the treatment of stage I, stage II, stage III, and stage IV pressure ulcers in adult patients. The authors compared the therapeutic effects of hydrocolloids and alternative dressings in the healing of pressure ulcers.

## 2. Methods

### 2.1. Search Strategy

To identify original studies, two independent authors (A.C. and M.K.) searched the databases of PubMed, Embase, and Cumulative Index to Nursing and Allied Health Literature (CINAHL) from their inception until 20 April 2020 for studies in English comparing the efficacy of hydrocolloid dressings with other dressings regarding the healing of ulcers. For the purposes of this review, a pressure ulcer was defined as a localized injury to the skin and/or underlying tissue, primarily caused by prolonged pressure and the associated tissue hypoxia or shear on the skin [1]. 

The following search strings with medical subject headings were used for PubMed, Embase, and CINAHL, listed in Table 1. 

Apart from the electronic search, a manual review of reference lists from existing meta-analyses and relevant reviews was performed.

### 2.2. Inclusion and Exclusion Criteria

The inclusion criteria were as follows:

- clinical trials comparing the efficacy of hydrocolloid and alternative dressings (simple dressings, dressings according to the British National Formulary classification);
- studies in adults (>18 years old);
- the presence of at least one pressure ulcer (stage I, II, III, or IV), with no restrictions on the type of pressure ulcer classification scale [10];
- various healthcare settings (outpatient clinics, home care agencies; acute-care facilities, family homes, long-term care nursing homes, geriatric hospital wards, rehabilitation centers, palliative care centers).

We excluded studies comprising fewer than ten participants.

### 2.3. Study Selection

At first, two reviewers independently evaluated the titles and abstracts of the articles searched in terms of significance, and then they independently checked the full texts of all potentially eligible studies for inclusion criteria. Disputes were resolved through discussion between two reviewers and, if necessary, a senior author was involved.

### 2.4. Data Extraction and Analysis

Two reviewers independently extracted data from included studies. The following data were abstracted: basic research features (e.g., country, funding sources), characteristics of participants (e.g., average age, proportions of participants by sex), features of pressure ulcers (e.g., stage, size, location), features of dressings used for pressure ulcers (e.g., type of dressing, frequency of use), evaluation of dressing effectiveness (e.g., wound healing time, unhealed wounds, or wounds whose condition worsened). Whenever the data were missing, the authors were contacted via email. In the case of Hondé et al.’s study [12], we were not able to verify the authors’ email addresses. 

The data extraction was carried out in line with PRISMA guidelines. The main result of this meta-analysis was regarding the effectiveness of hydrocolloids compared to the other therapeutic interventions (including other dressings) used in the treatment of pressure ulcers. To assess this, the number of pressure ulcers cured was analyzed. This result was taken into account either as the percentage of participants whose pressure ulcers were healed at the last control checkpoint (or a predetermined time point) or as the percentage of all persons successfully treated with pressure ulcers. Next, the incidence of pressure ulcers among the participants was assessed. In the analysis, the number of pressure ulcers was determined, taking into account their location and stage according to the pressure ulcer classification scale chosen by the papers’ authors. The final stage of the study was to assess the healing time of the wound, the frequency of dressing changes, and the duration of dressing wear. 

### 2.5. Assessment of the Risk of Bias in the Included Studies

The risk of bias in the included studies was assessed using a standardized critical appraisal instrument developed by the Cochrane Collaboration [13], which covers the following seven evaluation aspects: random sequence generation, allocation concealment, blinding of participants and personnel, blinding of outcome assessment, incomplete outcome data, selective reporting, and other sources of bias. The bias was presented as the number of points of low risk of bias assessment. 

### 2.6. Statistical Analysis

Random-effect meta-analysis of data from three or more studies was performed using meta-analysis software (Comprehensive Meta-Analysis V3, Biostat, NJ, USA [14]). Heterogeneity was assessed by means of chi-square tests of homogeneity. All analyses were two-tailed. Alpha was equal to 0.05. 

For continuous outcomes, we analyzed the pooled standardized mean difference (SMD) in endpoints using observed cases (OC) data. For nominal outcomes, the pooled risk ratio (RR) was calculated using OC data. The extent of asymmetry in funnel plots was detected using Egger’s tests.

Ethical approval and informed consent: not applicable.

## 3. Results

### 3.1. Search Results

**Figure 1:**
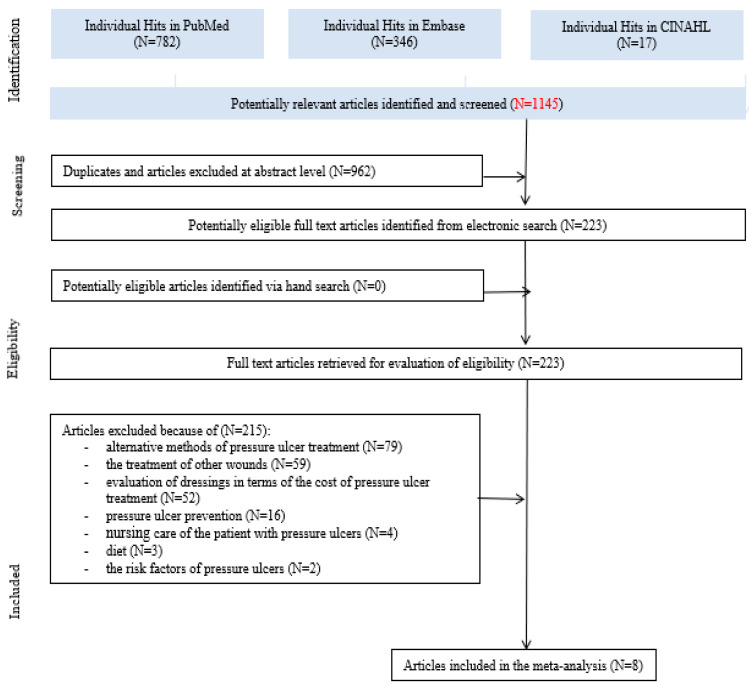
A flow diagram of the included and excluded studies.

Initially, a total of 1145 records were identified. After the first round of evaluation, we qualified a total of 223 for further full-text verification, of which eight were finally included in the present meta-analysis (Figure 1). In total, we excluded 215 articles. The major reasons were as follows: alternative methods of pressure ulcer treatment—e.g., larval therapy, negative pressure wound therapy (NPWT) (*n* = 79); the treatment of other wounds, such as diabetic foot and shin ulcers (*n* = 59); the evaluation of dressings in terms of the cost of pressure ulcer treatment (*n* = 52); pressure ulcer prevention (*n* = 16); nursing care of the patient with pressure ulcers (*n* = 4); diet (*n* = 3); and the risk factors for pressure ulcers (*n* = 2). 

The present meta-analysis included eight studies published in 1987–2019 [12,15,16,17,18,19,20,21]. The studies were conducted in Europe (*n* = 4) [12,15,17,20], West Asia (*n* = 1) [19], and North America (*n* = 3) [16,18,21] (Table 2).

### 3.2. Characteristics of the Included Studies

Three studies were non-industry-funded [17,19,20]. Three studies were industry-funded [12,15,16]. In two studies, no sources of funding were indicated [18,21] (Table 2).

There were 679 patients included, with male predominance (*n* = 349, 51.4%). The sizes of the study groups ranged from 34 to 169 people. The mean age of the participants for seven studies (except that of Bale et al. [15] was) 77.9 (SD 13.8) years (Table 2).

#### 3.2.1. Characteristics of Pressure Ulcers

Pressure ulcers were assessed using the Braden Scale (*n* = 3) [16,17,21], the Pressure Ulcer Scale for Healing (PUSH) (*n* = 1) [17], the Norton Scale (*n* = 1) [12], the NPUAP classification (*n* = 1) [19], the Shea Classification of Pressure Ulcers (*n* = 1) [12,19], the Stirling’s Pressure Ulcer Severity Scale (*n* = 1) [15], and the Torrance Scale (*n* = 1) [20].

Most researchers used one scale for the classification of pressure ulcers [15,16,20,21]. Some researchers evaluated pressure ulcers using several tools [12,17,19]. In one study, a pressure ulcer assessment scale was not indicated [18].

Three studies described the treatment of infected pressure ulcers [12,18,20]. The rest of the articles did not include information on the treatment of infected or uninfected pressure ulcers [15,16,17,19,21]. 

In the studies analyzed, a total of 737 pressure ulcers were evaluated. The most common locations of pressure ulcers were the area of the tailbone (*n* = 300) [12,15,16,17,18,19,20,21] and the heel/foot (*n* = 135) [12,15,16,17]. Locations where pressure ulcers were found less often were the buttock area (*n* = 80) [16,17,19,20] and hips (*n* = 51) [12,15,17,18] (Table 3).

The average area of pressure ulcers for four studies [16,19,20,21] was 7.13 cm^2^ (SD 12.5). In one study [15], the area of the wound had been divided into three categories: < 5 (*n* = 24), 5–10 (*n* = 12); 10–20 (*n* = 13), and > 20 cm^2^ (*n* = 11) (Table 3).

#### 3.2.2. Characteristics of Interventions 

The studies compared the effectiveness of hydrocolloids [12,15,16,17,18,19,20,21] with the effectiveness of a polyurethane foam dressing [15], a transparent absorbent acrylic dressing [16], a hydrocellular dressing [17], a mesh-gauze WDDs (wound and debridement dressing; wet-to-dry dressing)/simple dressing [18,19], a copolymer membrane [12], a lyofoam [20], a radiant-heat dressing device [21]. One of the studies [19] compared three types of dressings: hydrocolloids, simple dressings, and adhesive and phenytoin cream. The data selected for meta-analysis concerned two groups of dressings: the study group consisted of patients using hydrocolloids, and the control group comprised of those using other types of dressings (Table 2).

#### 3.2.3. Results of Interventions 

Not all studies reported a wound-healing rate, and the frequency of dressing changed. In five studies, adverse events were described. Only two studies indicated that hydrocolloids were superior to other dressings in some respects [18,19] (Table 4).

### 3.3. The Risk of Bias Assessment

Analysis of the risk of bias assessment demonstrated that only one study was high quality [19]. The risk of bias for all included studies is shown in Table 5.

### 3.4. Meta-Analysis

**Figure 2:**
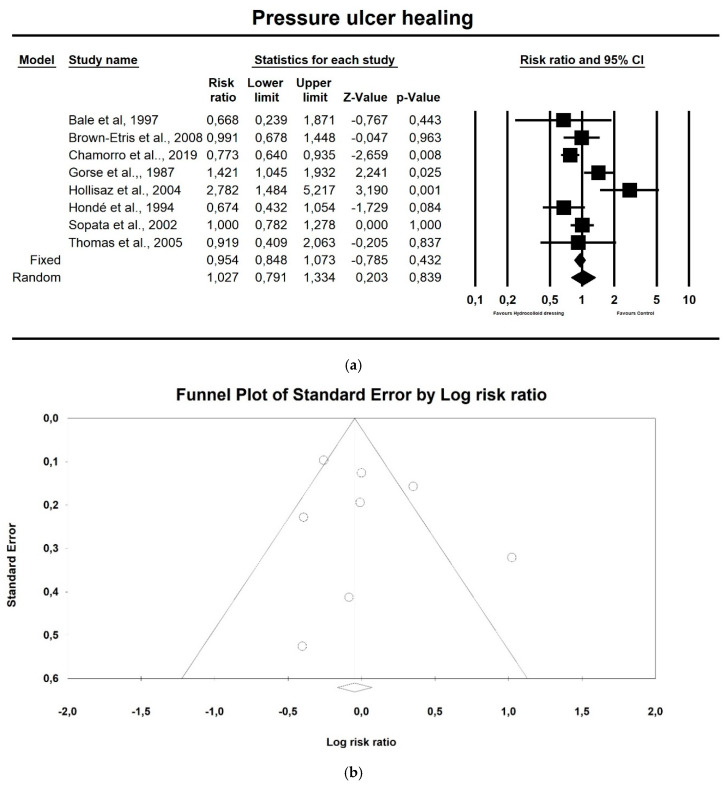
(**a**) The effect size for the pressure ulcer healing rate when using hydrocolloid vs. alternative dressings. Q = 25.272, df (Q) = 7, *p* = 0.001, I squared = 72.301; (**b**) Funnel plot for pressure ulcer healing (RR) in the present meta-analysis.

Regarding pressure ulcer healing, hydrocolloid dressings were not superior to control therapeutics, as shown in Figure 2a (*p* = 0.839; Z = 0.203; CI 95%: 0.791–1.334). Egger’s test did not indicate publication bias, as shown in Figure 2b (t value = 0.779, *p* = 0.465).

**Figure 3:**
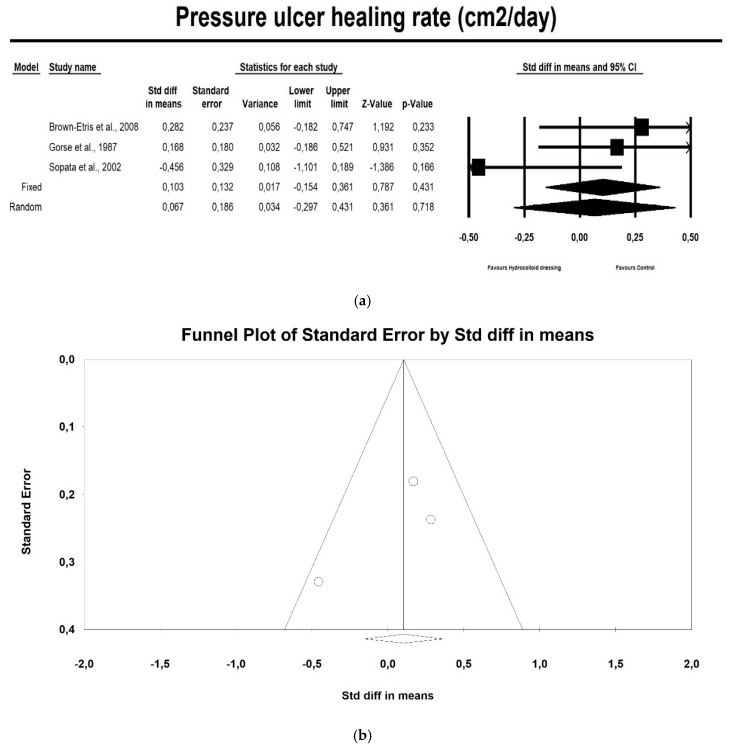
(**a**) The effect size for the pressure ulcer healing rate (cm^2^/day) when using hydrocolloid vs. alternative dressings. Q = 3.588, df (Q) = 1, *p* = 0.166, I squared = 44.25. (**b**) Funnel plot for the pressure ulcer healing rate (SMD) in this meta-analysis.

Hydrocolloids were not associated with a higher healing rate compared with control therapeutics, as shown in Figure 3a (*p* = 0.718; Z = 0.361; OR: 0.067; CI 95%: 0.297–0.431). Egger’s test did not indicate publication bias, as shown in Figure 3b (t value = 1.198, *p* = 0.442).

**Figure 4:**
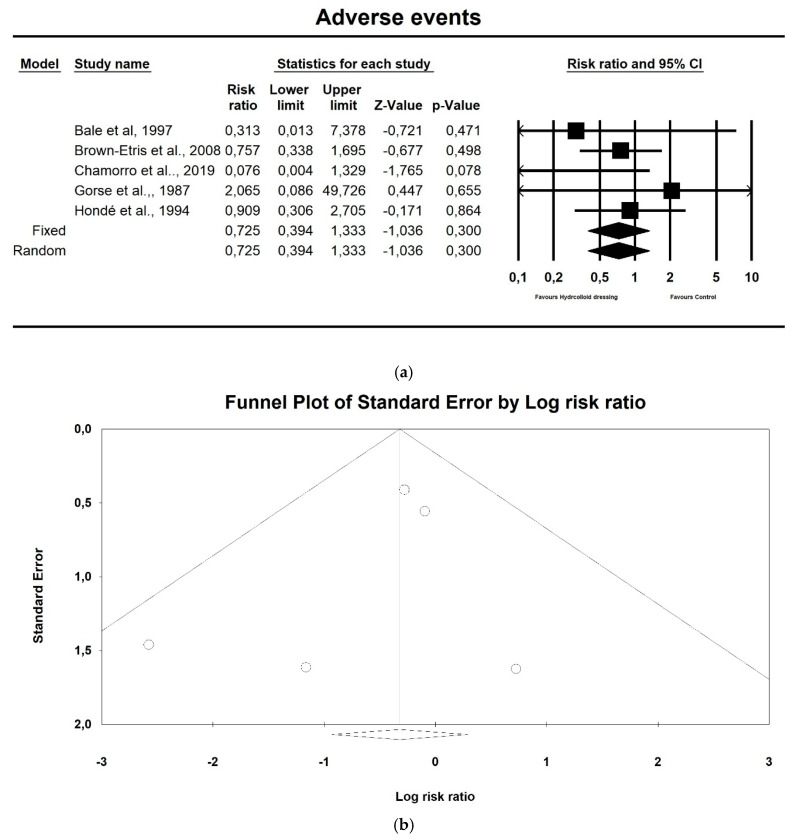
(**a**) The effect size for adverse events when using hydrocolloids vs. controls. Q = 3.251, df (Q) = 4, *p* = 0.517, I squared = 0.0. (**b**) Funnel plot for adverse events (RR) in the present meta-analysis.

Hydrocolloids did not reduce the incidence of adverse events compared to controls, as shown in Figure 4a (*p* = 0.300; Z = −1.036; OR: 0.067; CI 95%: 0.394–1.333). Egger’s test did not indicate publication bias, as shown in Figure 4b (t value = 0.834, *p* = 0.465).

## 4. Discussion

### 4.1. Main Findings

The main aspect taken into account in this meta-analysis was the effectiveness of hydrocolloids compared with other therapeutic interventions (including other dressings) used in pressure ulcer treatment. Eight studies were selected for meta-analysis [12,15,16,17,18,19,20,21], because they presented the use of hydrocolloids in comparison with alternative dressings.

Hydrocolloid dressings were not associated with a higher healing rate and were not superior to control therapeutics. The results are very interesting as hydrocolloids have until recently been regarded as the gold standard in the treatment of pressure ulcers in clinical practice, since they have most features of the so called ‘ideal dressing’. However, hydrocolloid dressings have both advantages and disadvantages. The advantage is that hydrocolloid dressings are made of non-adherent, high density, waterproof, highly absorbent materials, easily removed by saline or sterilized water without any pain. Nevertheless, such dressings have a diverse antimicrobial activity-based structure, are volumetrically unstable, and are not intended for heavily exudating wounds. In addition, dextran hydrocolloid delays healing, is impermeable to gases, and can cause an unpleasant odor after removal of the dressing [22].

### 4.2. Differences between Ours and Other Published Studies

Our findings are different from the results of other meta-analyses. A systematic review of the use of hydrocolloids in pressure ulcer treatment [23] demonstrated their greater effectiveness compared to gauze dressings, especially in the context of absorption capacity, the time needed to change a dressing, pain during dressing changes, and side effects. However, they were significantly less effective than alginates, polyurethane dressings, contactless layers, topical enzymes, and biosynthetic dressings.

Another meta-analysis [24] confirmed the greater efficacy of hydrocolloids but failed to confirm the advantages of other advanced dressings over conventional ones. The meta-analysis conducted by Huang et al. [25] showed that using hydrocolloids, foam dressings, and film dressings is more effective than standard care for the prevention of pressure ulcers in hospitalized patients. 

In turn, the results of the systematic review and meta-analysis by Pott et al. [26] were not sufficient to clearly determine whether the effectiveness of hydrocolloids is higher than that of alternative dressings.

### 4.3. Strenghts and Limitations

The main advantage of our systematic review with meta-analysis is that it concerns the assessment of the effectiveness of hydrocolloid dressings in the treatment of adult patients with stage I, II, III, and IV pressure ulcers, which is of particular importance in the context of aging societies. In this group of patients, preventive and therapeutic approaches in dealing with pressure ulcers are a big challenge. Another advantage of our study is the use of reliable data extraction instruments based on the guidelines contained in the PRISMA protocol. Moreover, the assessment of the risk of bias in the included studies was conducted using a standardized critical appraisal instrument developed by the Cochrane Collaboration.

The study limitation cannot be omitted in this part of the work. All publications included in this meta-analysis adopted various inclusion/exclusion criteria and small samples. Moreover, the duration of individual studies and observation periods varied widely. Moreover, the studies analyzed involved many uncontrolled variables (patients’ age, underlying diseases and comorbidities, nutritional status, medications used, medical history, pressure ulcer history, causes, sizes and locations of pressure ulcers, other methods of pressure ulcer treatment, length of stay in healthcare setting) that could have affected the final results of these studies. Not every study included in this meta-analysis assessed the risk factors for pressure ulcers separately for the study group and the control group, which makes it difficult to determine whether they were similar in both groups. Furthermore, the studies analyzed were carried out in various healthcare settings, which could have influenced the choice of the type of dressing, depending on the state of the wound, the patient’s clinical condition, comfort, and overall quality of life, as well as pressure ulcer treatment methods used so far, availability of the dressing, dressing wear time, the medical staff’s experience and knowledge of pressure ulcer treatment, and the overall cost of the treatment used. In two of the studies, the source of funding was not indicated, three studies were industry funded, and three studies were non-industry funded; hence, it is difficult to determine whether the means of financing had any impact on the outcomes of the research. Most studies did not provide details of randomization. Moreover, only one study had a high risk of bias.

Ultimately, all the factors discussed above could increase the margin of error and reduce the precision of the results obtained and thus directly contribute to the failure to show statistically significant differences between the studied dressings.

### 4.4. Implications for Current Practice and Future Research

Hydrocolloid dressings have been widely used in clinical practice since the early 1980s. Differing in structure and function, they still meet the criteria for an absorbent dressing that adheres well and protects the wound from external factors and provides a warm and moist environment that promotes better wound healing. By absorbing water and low molecular weight ingredients, hydrocolloids form a characteristic gel that stimulates the immune system and reduces the effects of bacterial colonization [27]. Clinical studies of hydrocolloids on more than 2000 pressure ulcers have shown a significantly lower incidence of infections than in the case of other treatments [28,29]. These are the most common advantages of using hydrocolloid dressings compared to conventional methods. 

Since the first use of hydrocolloids in clinical practice, their structure and function have been subject to innovative modifications. Nevertheless, the main limitation of traditional hydrocolloid dressings, often pointed out by clinicians, is their opacity, i.e., the inability to visualize the wound, exudate, and skin around the dressing, potentially leading to premature removal of the dressing in order to observe the wound. There are also many other limitations of this type of dressing—for example, a relatively high-edge profile of some non-bordered versions; limited conformability; residue left in the wound and on the periwound skin from the formation of a liquefied gel; and a distinctive, unpleasant odor after absorption of wound drainage [16,30,31,32]. These factors can reduce the effective wearing time of many hydrocolloid dressings, which directly affects their effectiveness, the time and cost of treatment, as well as the quality of care and the quality of patients’ lives. 

Currently available evidence is insufficient to assess whether any dressing or topical treatment increases the likelihood of pressure ulcer healing more than others and to assess whether there is a negative relative effect on wound healing or not. None of the interventions appear to heal more wounds than others [11]. For this reason, according to some researchers, when choosing a suitable dressing, clinicians should take into account wound symptoms, clinical experience, patient preference, and the cost of intervention [11,33].

Westby et al. assert that there is no high-quality scientific evidence for a beneficial effect of individual wound dressings or local treatment on wound healing, even when compared to basic dressings. This lack of evidence is worrying because of the high personal and health service burden of pressure ulcers, as well as the numerous potential participants who could be invited to take part in trials. The network meta-analysis by Westby et al. reveals the generally poor quality of randomized controlled trials of pressure ulcer dressings, suggesting that there is a need for radical improvement in planning and testing in this field [11].

According to Walker et al., where trials are conducted, good practice guidelines on their design, implementation, and reporting should be followed. Further reviews are underway to synthesize evidence regarding the effects of other dressings on pressure ulcer treatment. It would then be useful to conduct further evidence synthesis to help make decisions on the choice of pressure ulcer dressings across all dressing options [33].

## 5. Conclusions

Based on the results of our systematic review with meta-analysis, the following conclusions were drawn regarding pressure ulcer treatment using hydrocolloid dressings: the evidence from this meta-analysis is insufficient to conclude that hydrocolloids are more effective in pressure ulcer treatment in adult patients than alternative dressings. There is a need for further research to confirm or reject this hypothesis and demonstrate the real benefit of special dressings. Furthermore, the results of our research are not a basis for changing clinical practice in relation to the use of hydrocolloids in pressure ulcer treatment. 

## Figures and Tables

**Table 1:** Search strings with medical subject headings were used for PubMed, Embase, and CINAHL.

Database	Search Strings with Medical Subject Headings
PubMed	(‘pressure ulcer’ [MeSH Terms] OR (‘pressure’ [All Fields] AND ‘ulcer’ [All Fields]) OR ‘pressure ulcer’ [All Fields] OR ‘decubitus’ [All Fields]) AND (‘adult’ [MeSH Terms] OR ‘adult’ [All Fields]) AND (‘bandages’ [MeSH Terms] OR ‘bandages’ [All Fields] OR ‘dressing’ [All Fields]) AND (‘therapy’ [Subheading] OR ‘therapy’ [All Fields] OR ‘treatment’ [All Fields] OR ‘therapeutics’ [MeSH Terms] OR ‘therapeutics’ [All Fields]). The following search terms with medical subject headings (MeSH–bold font)
Embase	(‘decubitus’/exp OR ‘bed sore’ OR ‘bedsore’ OR ‘decubital ulcer’ OR ‘decubital ulcus’ OR ‘decubitus’ OR ‘decubitus ulcer’ OR ‘decubitus ulceration’ OR ‘decubitus ulcers’ OR ‘decubitus ulcus’ OR ‘decubus ulcer’ OR ‘pressure sore’ OR ‘pressure ulcer’ OR ‘sore, pressure’ OR ‘ulcer, pressure’ OR ‘ulcus decubitus’) AND (‘human’/exp OR ‘homo sapiens’ OR ‘human’ OR ‘human being’ OR ‘human body’ OR ‘human race’ OR ‘human subject’ OR ‘humans’ OR ‘man (homo sapiens)’) AND (‘adult’/exp OR ‘adult’ OR ‘adults’ OR ‘grown-ups’ OR ‘grownup’ OR ‘grownups’) AND (‘wound dressing’/exp OR ‘amd telfa’ OR ‘adaptic (device)’ OR ‘adaptic touch’ OR ‘algisite’ OR ‘aquacel’ OR ‘aquacel-ag’ OR ‘askina calgitrol’ OR ‘atrauman’ OR ‘autologel’ OR ‘biopatch’ OR ‘biostep’ OR ‘curasorb’ OR ‘cutilin’ OR ‘cutimed sorbact’ OR ‘drymax’ OR ‘eclypse (device)’ OR ‘excellagen’ OR ‘flivasorb’ OR ‘graftskin’ OR ‘hemcon’ OR ‘hemcon bandage pro’ OR ‘hemcon dental dressing pro’ OR ‘hemcon nasal plug’ OR ‘hemcon strip pro’ OR ‘jaloskin’ OR ‘kerramax’ OR ‘leukomed’ OR ‘leukomed sorbact’ OR ‘medihoney’ OR ‘mepitel’ OR ‘mepore’ OR ‘mesorb’ OR ‘opsite (device)’ OR ‘primatrix ag’ OR ‘primapore’ OR ‘promogran’ OR ‘quickclot acs’ OR ‘seasorb’ OR ‘silvercel’ OR ‘sorbion’ OR ‘steri-strips’ OR ‘suprathel’ OR ‘surfasoft’ OR ‘telfa’ OR ‘veloderm’ OR ‘zetuvit’ OR ‘askina sorb’ OR ‘biobrane’ OR ‘dressing, wound’ OR ‘oasis (device)’ OR ‘wound dressing’ OR ‘wound dressing agent’) AND (‘therapy’/exp OR ‘combination therapy’ OR ‘disease therapy’ OR ‘disease treatment’ OR ‘diseases treatment’ OR ‘disorder treatment’ OR ‘disorders treatment’ OR ‘efficacy, therapeutic’ OR ‘illness treatment’ OR ‘medical therapy’ OR ‘medical treatment’ OR ‘multiple therapy’ OR ‘polytherapy’ OR ‘somatotherapy’ OR ‘therapeutic action’ OR ‘therapeutic efficacy’ OR ‘therapeutic trial’ OR ‘therapeutic trials’ OR ‘therapeutics’ OR ‘therapy’ OR ‘therapy, medical’ OR ‘treatment effectiveness’ OR ‘treatment efficacy’ OR ‘treatment, medical’). The following search terms with medical subject headings (MeSH–bold font)
CINAHL	(‘pressure ulcer’ OR ‘bedsore’ OR ‘decubitus ulcer’ OR ‘pressure sore’) AND (‘dressings’ OR ‘bandages’) AND (‘treatment’ OR ‘intervention’ OR ‘therapy’) AND (‘adults’ OR ‘adult’ OR ‘aged’ OR ‘elderly’)

**Table 2:** Characteristics of the included studies.

Characteristics of Included Studies											
No.	Reference (Localization)	Age (years) (Mean ± SD)	Subjects/Males (*n*)	Healthcare Setting	Pressure Ulcers at the Beginning of the Study SG/CG (*n*)	Healed Pressure Ulcers SG/CG (*n*)	Healed Pressure Ulcers (*n*)/A Total Number of Pressure Ulcers (*n*)	Length of Follow up	Source of Funding	The Types of Dressings	
										SG	CG
1.	Bale et al., 1997 (the UK)	nd (median: SG: 74; CG: 73)	60/27	5 centers in the UK	31/29	5/7	12/60	Up to 4 weeks (30 days).	Financial support from Smith and Nephew—industry funded.	A hydrocolloid dressing	A polyurethane foam dressing
2.	Brown-Etris et al., 2008 (the USA, Canada)	SG: 72.7 (18.6); CG: 78.3 (14.7)	72/32	A variety of healthcare settings, including extended-care facilities, outpatient wound care clinics, and home care agencies	37/35	22/21	43/72	Up to 8 weeks (56 days).	Grant from 3M Company (manufacturers of Tegaderm)—industry funded.	A hydrocolloid dressing (DuoDERM CGF, ConvaTec, ER Squibb & Sons, Princeton, NJ)	A transparent absorbent acrylic dressing (3M Tegaderm Absorbent Clear Acrylic Dressing)
3.	Chamorro et al., 2019 (Spain)	SG: 83.3 (8.7); CG: 79.2 (13.3)	169/71	Primary care centers and ling-term care institutions	85/84	54/69	123/169	Up to 8 weeks	Grant from the Ministry of Economy and Competitiveness, Carlos III Institute (ISCIII), as well as with financial support from the Health Promotion and Preventive Activities- Primary Health Care Network, the Ministry of Health ISCIII-RETIC), and the European Union Regional Development Funds – non-industry funded.	A hydrocolloid dressing (VARIHESIVE^®^ GEL CONTROL (Convatec)	A hydrocellular dressing (ALLEVYN Adhesive^®^ (Smith & Nephew)
4.	Gorse et al., 1987 (the USA)	SG: 72.0 +/− 12.8; CG: 68.4–13.5	52/52	The Huntington Veterans Administration Medical Center—acute-care facility	76/52	54/26	80/128	Approx. 11 weeks.	nd	A hydrocolloid dressing (Duoderm, Convatec, E.R. Squib and Sons Inc.)	A mesh-gauze wet-to-dry dressing (WDDs)
5.	Hollisaz et al., 2004 (Iran)	36.64 ± 6.04	83/83	Family homes or nursing homes	31/30	23/8	31/61	Approx. 8 weeks (2 months).	Grant from the Jaonbazan Medical and Engineering Research Center, the medical and research section of the official governmental body responsible for spinal cord injury (SCI) war victims—non-industry funded.	A hydrocolloid dressing	A simple dressing
6.	Hondé et al., 1994 (France)	general—82 yearsSG: 83.5 +/− 7.8 (years 64–101); CG: 80.4 +/− 8.2 (years 63-98);	168/47	Geriatric hospital wards	88/80	23/31	54/168	Up to 8 weeks.	Financial support from Synthelabo Recherche (manufacturers of Inerpan)—industry funded.	A hydrocolloid dressing, Comfeel™ (Coloplast)	A copolymer membrane, Inerpan™ (Synthélabo)
7.	Sopata et al., 2002 (Poland)	general: 58.6 +/− 15.51; SG: 58.7 +/− 14.11; CG: 58.5 +/− 16.92	34/16	Palliative Care Department at the University of Medical Sciences, Poznan, Poland	17/17	15/15	30/38	Up to 8 weeks.	This study was non-industry funded—declaration of interest: none.	A hydrogel dressing (Aquagel, Wytwórnia Opatrunków, Poland)	A polyurethane foam dressing Lyofoam (Seton, UK)
8.	Thomas et al., 2005 (the USA)	General—75.5 +/− 12.6; SG: 77.0 +/− 11.5; CG:74.1 +/− 13.8	41/21	Outpatient clinics, long-term care nursing homes, and a rehabilitation center	20/21	7/8	15/41	Up to 12 weeks	nd	A sterile control hydrocolloid dressing (Duoderm™ Convatec, Inc., Princeton, NJ) with or without a calcium alginate filler (Sorbasan,™ Smith-Nephew, Inc., Largo, FL)	A radiant-heat dressingdevice (Warm-Up™, Augustine Medical, Inc., Princeton, NJ)

Note: nd—no data; SG—study group; CG—control group; source: the authors’ own analysis.

**Table 3:** Characteristics of pressure ulcers in the included studies.

No.	Reference (Localization)	Characteristics of the Pressure Ulcers											
		Pressure Ulcers (*n*)	Stage (*n*)				Localization (*n*)						Baseline Size cm^2^ (Mean ± SD) (cm^2^ +/− SD)
			I	II	III	IV	Sacrum/ Coccyx	Foot/ Heel	Buttock	Ischium	Trochanter	Other	
	Bale et al., 1997 (the UK)	60	0	45	15	0	31	16	nd	nd	2	11	(sore area (cm^2^) >5: *n* = 24 (sore area (cm^2^) 5–<10: *n* = 12 (sore area (cm^2^) 10–<20: *n* = 13 (sore area (cm^2^) ≥ 20: *n* = 11
	Brown-Etris et al., 2008 (the USA, Canada)	72	0	45	27	0	22	8	14	12	nd	16	SG = 2.5 +/− 4.86 (cm^2^ +/− SD) CG = 1.5 +/− 1.69 (cm^2^ +/− SD)
	Chamorro et al., 2019 (Spain)	169	0	169	0	0	75	19	40	0	15	20	nd
	Gorse et al., 1987 (the USA)	128	0	107	21		56	nd	nd	22	29	21	nd
	Hollisaz et al., 2004 (Iran)	61	24	37	0	0	15	0	14	32	0	0	SG = 7.26 +/− 15.4 (cm^2^ +/− SD) CG = 10.27 +/− 15.32 (cm^2^ +/− SD)
	Hondé et al., 1994 (France)	168	0	nd	nd	nd	61	92*	0	0	5	10	SG = 6.85 cm^2^ CG = 8.99 cm^2^
	Sopata et al., 2002 (Poland)	38	0	12	26	0	17	nd	12	nd	nd	nd	SG = 8.28 +/− 13.90 (cm^2^ +/− SD) CG = 11.04 +/− 11.65 (cm^2^ +/− SD)
	Thomas et al., 2005 (the USA)	41	0	0	22	19	23 **	nd	nd	9 **	nd	9 **	SG = 12.1 +/− 18.2 (cm^2^ +/− SD) CG = 11.0 +/− 9.5 (cm^2^ +/− SD)

Note: nd—no data; SG—study group; CG–control group; source: the authors’ own analysis; *—the number deduced due to an error in the text of the publication; **—the numbers calculated from percentage values given in the text of the publication.

**Table 4:** Characteristics of interventions in the included studies.

No.	Reference (Localization)	Wound Healing (Rate)		Frequency of Dressing Change Dressing Wear Time		Adverse Events (*n*)		Conclusion
		SG	CG	SG	CG	SG	CG	
	Bale et al., 1997 (the UK)	nd	nd	Mean wear times: SG: 3.2 days; The maximum wear time for an individual dressing was 11 days.	Mean wear times: CG: 3.8 days The maximum wear time for an individual dressing was 13 days.	0	1 (skin rash)	SG and CG are easy and convenient to apply; absorbency and ease of removal were significantly better with CG than SG; wear times were similar.
	Brown-Etris et al., 2008 (the USA, Canada)	Linear healing rate, cm/wk Mean (SD): 0.12 (0.136).	Linear healing rate, cm/wk Mean (SD): 0.10 (0.205).	Mean (SD) wear time was: 4.7 (2.29) days.	Mean (SD) wear time was: 5.7 (2.55) days.	8 None of the adverse events were related to the study dressings under evaluation.	10 None of the adverse events were related to the study dressings under evaluation.	Performance results favored the CG over the SG as standard treatment for stage II and shallow stage III pressure ulcers.
	Chamorro et al., 2019 (Spain)	nd	nd	The dressing was changed every 7 days.	The dressing was changed every 7 days.	0	6 (infection, erythema, dressing hypersensitivity)	CG were superior to SG in terms of healing at 8 weeks and time required for healing. These two dressings had similar safety profiles.
	Gorse et al., 1987 (the USA)	Completely healed Rate of decrease, cm^2^/d: 0.72 ± 1.22 Days to resolution: 10.0 ± 10.5	Completely healed Rate of decrease, cm^2^/d: 0.55 ± 0.59 Days to resolution: 8.7 ± 6.2	The dressing was changed routinely every four days or more frequently if the membrane became contaminated with stool, became nonocclusive, or if signs and symptoms of systemic infection developed.	The dressings were changed every eight hours.	1 (infection)	0	SG regimen was more efficacious even in a subgroup of patients who did not receive adequate nutritional support during treatment. Adequate nutritional support during the study was associated with better healing in both SG and CG.
	Hollisaz et al., 2004 (Iran)	*n* (%) The completion of healing, regardless of location and stage: 23/31 (74.19%). Completion of healing of stage I ulcers: 11/13 (85%). Completion of healing of stage II ulcers: 12/18 (67%).	*n* (%) The completion of healing, regardless of location and stage: 8/30 (26.66%). Completion of healing of stage I ulcers: 5/11 (45%). Completion of healing of stage II ulcers: 3/19 (16%).	Twice a day.	Twice a day.	0	0	SG is the most effective method investigated for treating stage I and II pressure ulcers in young paraplegic men.
	Hondé et al., 1994 (France)	The median healing time was 38 (range 11–63) days.	The median healing time was 32 (range 13–59) days.	nd	nd	6 (infection)	6 (infection)	GK is easy to use, safeguards the healing process, and is of particular value in the management of pressure sores.
	Sopata et al., 2002 (Poland)	Rate of healing (cm^2^/day): 0.67 ± 0.37 cm^2^/day (grade II) and 0.31 ± 0.21 cm^2^/day (grade III). “Improved” ulcers (grade III only) healed at 0.27 ± 0.11 cm^2^/day. Treatment times (days):Medium time: 20.10 ± 14.70 (*n* = 20)	Rate of healing (cm^2^/day): 1.23 ± 1.33 cm^2^/day (grade II) and 0.44 ± 0.27 cm^2^/day (grade III). “Improved” ulcers (grade III only) healed at 0.70 ± 0.63 cm^2^/day. Treatment times (days): Medium time: 25.77 ± 14.15 (*n* = 18)	Dressings were changed according to clinical need.	Dressings were changed according to clinical need.	0	0	There was no statistical difference between SG and CG in efficacy, healing rates, and treatment times.
	Thomas et al., 2005 (the USA)	*n* (%): 7 (44%) with complete healing of their pressure ulcer.	*n* (%): 8 (57%) with complete healing of their pressure ulcer.	The dressing was changed every 7 days or when the occlusive seal was broken.	The dressing was changed every 7 days or when the occlusive seal was broken.	nd (adverse events and serious adverse events were assessed at each weekly visit).	nd (adverse events and serious adverse events were assessed at each weekly visit).	There was no statistical difference between SG and CG. However, at almost all points along the healing curve, the proportion not healed was higher in SG.

Note: nd—no data; SG—study group; CG—control group; source: the authors’ own analysis.

**Table 5:** Assessment of the risk of bias in the included studies.

Reference (Localization)	Random Sequence Generation (Selection Bias)	Allocation Concealment (Selection Bias)	Blinding of Participants and Personnel (Performance Bias)	Blinding of Outcome Assessment (Detection Bias)	Incomplete Outcome Data	Selective Reporting (Reporting Bias)	Other Sources of Bias	Number of Low Risk of Bias Assessments
Bale et al., 1997 (the UK)	?	H	H	H	H	L	L	2
Brown-Etris et al., 2008 (the USA, Canada)	?	?	H	H	L	L	L	3
Chamorro et al., 2019 (Spain)	?	H	H	L	H	H	H	1
Gorse et al., 1987 (the USA)	?	H	?	?	L	L	H	2
Hollisaz et al., 2004 (Iran)	L	L	L	L	L	L	?	6
Hondé et al., 1994 (France)	?	?	H	H	H	H	?	0
Sopata et al., 2002 (Poland)	?	?	?	?	L	L	H	2
Thomas et al., 2005 (the USA)	?	?	H	H	H	L	L	2

Note: L—low risk of bias; H—high risk of bias; ?—unclassified risk of bias; source: the authors’ own analysis.
